# Supplementary material for: Resonantly exited precession motion of three-dimensional vortex core in magnetic nanospheres
Source: Sci Rep. 2015 Jun 16;5:11370. doi: 10.1038/srep11370 (PMC4468820; doi:10.1038/srep11370)
Supplement: Supplementary Information [file srep11370-s2.pdf]

Resonantly exited precession motion of three-dimensional vortex core in magnetic nanospheres

Sang-Koog Kim[1,*], Myoung-Woo Yoo[1], Jehyun Lee[1,+], Ha-Youn Lee[1], Jae-Hyeok Lee[1], Yuri Gaididei[2], Volodymyr P. Kravchuk[2], and Denis D. Sheka[3]

[1] National Creative Research Initiative Center for Spin Dynamics and Spin-Wave Devices, Nanospinics Laboratory, Research Institute of Advanced Materials, Department of Materials Science and Engineering, Seoul National University, Seoul 151-744, South Korea

[2] Bogolyubov Institute for Theoretical Physics, 03680 Kiev, Ukraine

[3] Taras Shevchenko National University of Kiev, 01601 Kiev, Ukraine

[*] sangkoog@snu.ac.kr

[+] Present address: Center of Semiconductor Research & Development, Gyeonggi-do 445-701, South Korea

Supplementary Movie

Supplementary movie presents a dynamic motion of the precession of a vortex core in the sphere of $2R$ = 80 nm, as driven by H_{DC} = 100 Oe. The red, white and orange arrows represent the individual directions of the core magnetization, the local curling magnetization and the externally applied field, respectively.
